# Etv2 transcriptionally regulates *Yes1* and promotes cell proliferation during embryogenesis

**DOI:** 10.1038/s41598-019-45841-5

**Published:** 2019-07-05

**Authors:** Bhairab N. Singh, Wuming Gong, Satyabrata Das, Joshua W. M. Theisen, Javier E. Sierra-Pagan, Demetris Yannopoulos, Erik Skie, Pruthvi Shah, Mary G. Garry, Daniel J. Garry

**Affiliations:** 1 Medicine Department and the Lillehei Heart Institute, University of Minnesota, Minneapolis, MN 55455 USA; 2 Department of Pediatrics, University of Minnesota, Minneapolis, MN 55455 USA; 3 Paul and Sheila Wellstone Muscular Dystrophy Center, University of Minnesota, Minneapolis, MN 55455 USA; 4 Stem Cell Institute, University of Minnesota, Minneapolis, MN 55455 USA

**Keywords:** Cell proliferation, Cell division

## Abstract

Etv2, an Ets-transcription factor, governs the specification of the earliest hemato-endothelial progenitors during embryogenesis. While the transcriptional networks during hemato-endothelial development have been well described, the mechanistic details are incompletely defined. In the present study, we described a new role for Etv2 as a regulator of cellular proliferation via *Yes1* in mesodermal lineages. Analysis of an Etv2-ChIPseq dataset revealed significant enrichment of Etv2 peaks in the upstream regions of cell cycle regulatory genes relative to non-cell cycle genes. Our bulk-RNAseq analysis using the doxycycline-inducible Etv2 ES/EB system showed increased levels of cell cycle genes including *E*2*f4* and *Ccne1* as early as 6 h following Etv2 induction. Further, EdU-incorporation studies demonstrated that the induction of Etv2 resulted in a ~2.5-fold increase in cellular proliferation, supporting a proliferative role for Etv2 during differentiation. Next, we identified *Yes1* as the top-ranked candidate that was expressed in *Etv2*-*EYFP*^+^ cells at E7.75 and E8.25 using single cell RNA-seq analysis. Doxycycline-mediated induction of Etv2 led to an increase in *Yes1* transcripts in a dose-dependent fashion. In contrast, the level of *Yes1* was reduced in *Etv2 null* embryoid bodies. Using bioinformatics algorithms, biochemical, and molecular biology techniques, we show that Etv2 binds to the promoter region of *Yes1* and functions as a direct upstream transcriptional regulator of *Yes1* during embryogenesis. These studies enhance our understanding of the mechanisms whereby Etv2 governs mesodermal fate decisions early during embryogenesis.

## Introduction

Transcriptional regulators, such as Mesp1and Etv2, and signaling pathways, including the Vegf1 and Shh pathways, are essential for the regulation of hemato-endothelial lineages during development^1–4^. Recent studies have demonstrated that Etv2 functions as a master regulator of hemato-endothelial specification during embryogenesis^2,5–7^. Etv2 is expressed transiently in primitive angioblasts and regulate lineage specification during embryogenesis^8^. Global knockout of *Etv2* results in embryonic lethality by E9.5 due to the complete absence of hemato-endothelial lineages^2,6^. Etv2 transactivates multiple targets including *miR*-*130a*, *Tie2* and *Lmo2* to regulate the hematoendothelial program^1,2,8^. Similarly, the interactions of Etv2 with Gata2 and FoxC2 have been shown to be important in the regulation of hemato-endothelial development^9,10^. Recently, we have shown coordination between Etv2 and Flt1-Flk1 signaling in the regulation of hemato-endothelial lineage differentiation during embryogenesis^11^. These studies suggest that interactions between transcription factors and signaling pathways determine hemato-endothelial cell fate. While the transcriptional and signaling networks in hematoendothelial development have been well described, the mechanistic details are incomplete.

Precise control of cell number is essential for proper development during embryogenesis^12,13^. The transcriptional effectors of Hippo signaling pathway, YAP and TAZ plays a critical role in controlling organ size and stem cell functions^12^. YAP (Yes Associated Protein) was first discovered as a binding partner of the Src-family tyrosine kinase, c-Yes (Yes1)^14^. Multiple kinases including: Src, Yes1 and Fyn, phosphorylates YAP or TAZ at the conserved tyrosine residue and regulate their roles as transcriptional activators^15^. The knockout of Src-family kinases result in embryonic lethality by E9.5 and are required to modulate extracellular signals^16,17^. Yes proto-oncogene 1 (Yes1) (a member of tyrosine kinase family) is highly expressed in the endothelial lineages^18,19^. Mice lacking Yes1 were found to show defective VEGF-induced vascular permeability supporting the hypothesis that Yes1 mediates an angiogenic response^17^. Similarly, homozygous deletion of YAP resulted in embryonic lethality by E8.5 due to defective yolk-sac vasculogenesis and cardiac abnormalities^20^. These studies support an important role for Yes1 and Hippo signaling in the endothelial lineages. The Yes1 protein consists of three domains including Src-homology (SH) 2 domain, SH3 domain and protein kinase domain. The Src-homology 3 (SH3) domain of Yes1 binds to the proline-rich region of YAP to promote YAP-mediated cellular survival and proliferation^21^. Recent studies have indicated that Yes1-induced tyrosine phosphorylation of YAP results in formation of a YAP-Tbx5-β-catenin complex to promote an anti-apoptotic process and proliferation^22^. These studies support the notion that Yes1 has a critical role in the regulation of YAP activity, however, the upstream regulators of Yes1 are not well described.

In the present study, using ChIPseq, ATACseq, bulk RNAseq and single cell RNAseq (scRNAseq) analyses, we demonstrate that Etv2 binds to the upstream regulatory regions of cell cycle genes. Our data demonstrate that Etv2 promotes cellular proliferation during embryonic development. Mechanistically, we demonstrate that Etv2 transcriptionally activates *Yes1* gene expression to regulate cellular proliferation.

## Results

### Etv2 binds to the upstream regulatory regions of cell cycle genes

**Figure 1:**
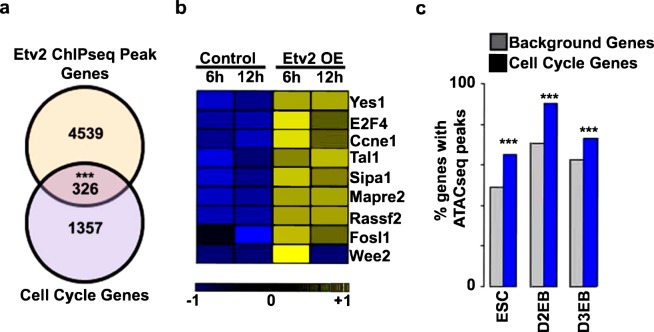
ChIPseq, ATACseq, and bulk RNAseq showed significant enrichment of cell cycle transcripts following induction of Etv2 in the ES/EB system. (**a**) Venn diagram of the overlap between genes associated with Etv2 ChIPseq peaks^25^ and genes annotated to the cell cycle GO-classification. The significance was confirmed using the Fisher Exact Test. (**b**) Heat map of bulk RNAseq analyses of the previously published datasets^10^ using *iHA*-*Etv2* ES/EBs showing increased expression of cell cycle genes in Dox-induced EBs relative to uninduced EBs. Note the increased expression of cell cycle genes following the induction of HA-Etv2 at both time points. (**c**) ATACseq analysis using *iHA*-*Etv2* ESCs, D2 EBs, and D3 EBs following Dox treatment for 24 h. Note that there was significantly (p < 0.001) higher percentage of ATACseq peaks within the cell cycle genes as compared to background genes in the Dox-treated samples (***p < 0.001).

**Figure 2:**
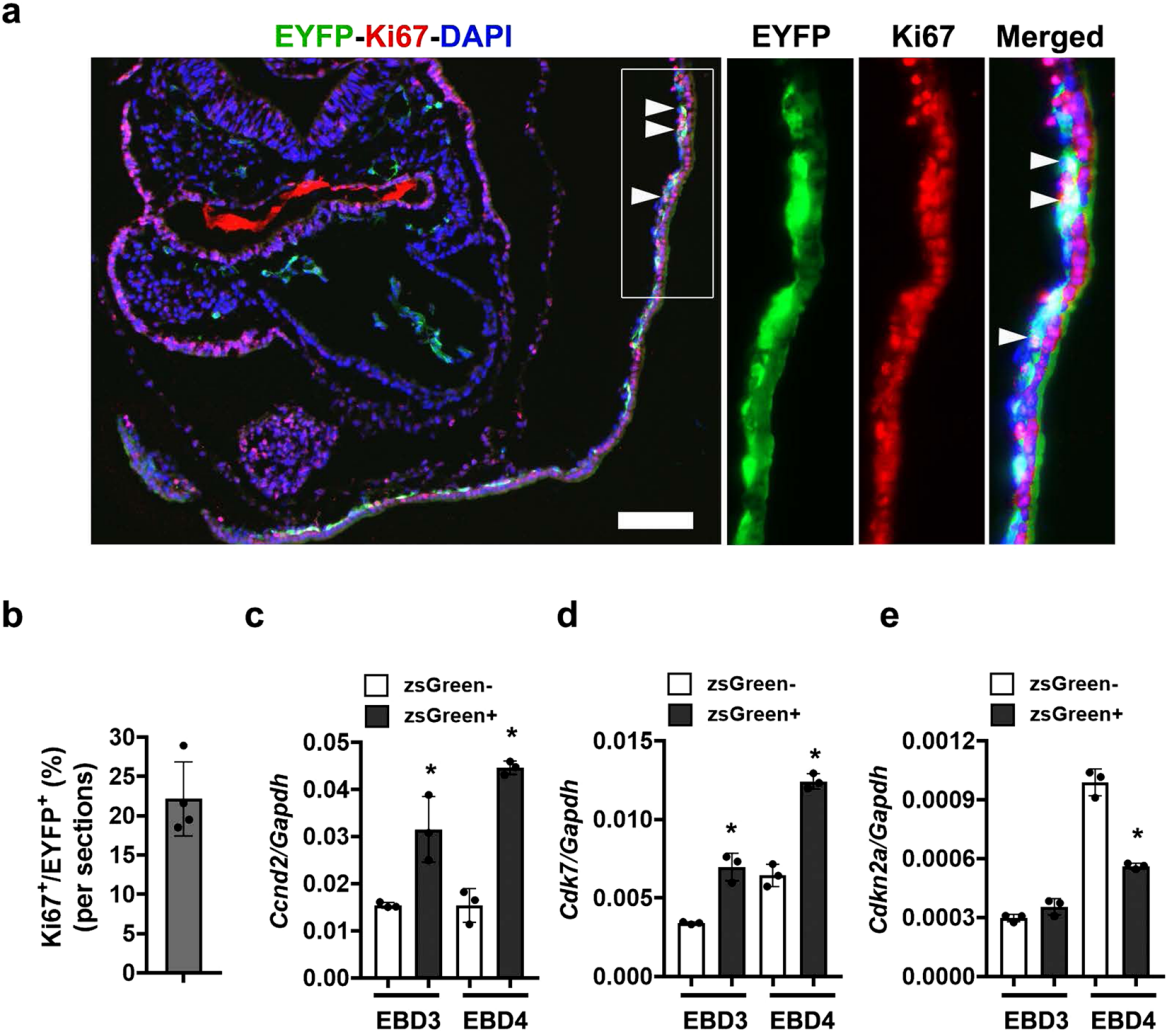
Etv2 is associated with cellular proliferation during embryogenesis. (**a**) Immunohistochemical analysis of transgenic *Etv2*-*EYFP* mouse embryos^5^ at E8.5. The boxed region is magnified and shown in the right panels. White arrowheads indicate EYFP^+^/Ki67^+^ double-positive cells. (**b**) Quantitative analysis of EYFP^+^ and Ki67^+^ cells in the Etv2-EYFP transgenic embryo sections. (**b**–**d**) qPCR analysis of cell cycle gene expression from zsGreen^−^ and zsGreen^+^ cells sorted from *Etv2*-zsGreen1-DR^11^ EBs on D3 or D4 of differentiation. Pro-proliferative cell cycle genes *Ccnd2* and *Cdk7*, showed increased expression in zsGreen^+^ cells, while expression of *Cdkn2a*, a cell cycle repressor, was decreased in zsGreen^+^ cells relative to zsGreen^−^ cells. Data are presented as mean ± SEM (n = 3 replicates; *p < 0.05).

Previous studies have demonstrated that *Etv2* mutants have altered mesodermal lineage specification^5,23,24^. To examine the potential role of Etv2 as a regulator of cellular proliferation, we analyzed a published ChIPseq datasets for Etv2 during embryoid body (EB) differentiation^25^. We obtained the cell cycle gene list using available database and the Gene Ontology (GO)-classification (GO:0007049), which includes both positive and negative regulators of the cell cycle. In this analysis, we identified multiple genes with associated Etv2 ChIPseq peaks. Our analysis of these data revealed significant overlap between GO-annotated cell cycle genes and those genes associated with Etv2 ChIPseq peaks, as compared to genes not associated with Etv2 ChIPseq peaks (Fig. 1a). We examined ± 5 kb upstream/downstream of the transcriptional start site (TSS) from the nearby genes as outlined in Supplemental Table 1. These results supported the hypothesis that Etv2 modulates cell proliferation through the regulation of cell cycle genes. To examine this hypothesis, we utilized our previously published bulk-RNAseq datasets^10^, obtained from differentiated mouse embryonic stem cells (ESCs) that inducibly overexpress (Dox-inducible) Etv2 (iHA-Etv2)^8^. We had performed bulk RNAseq analysis on day (D)3 EBs (D3 EBs) following the treatment with Dox (Etv2 OE) or vehicle control (−Dox) for 6 h or 12 h periods^10^. We examined the 6 h and 12 h time points following Dox treatment to identify direct downstream targets of Etv2. Consistent with the ChIPseq analysis, our analysis of the published bulk-RNAseq datasets^10^ showed multiple transcripts involved in the regulation of cell cycle progression, including *Yes1*, *E2f4*, *Ccne1*, which were upregulated following Dox-mediated induction of Etv2 (Fig. 1b and Supplemental Table 2). To determine whether induction of cell cycle transcripts by Etv2 overexpression were accompanied by enhanced chromatin accessibility, we undertook ATACseq analysis of chromatin accessibility changes in *iHA*-*Etv2* ESCs, D2 EBs and D3 EBs following treatment with (+Dox) or without Dox (−Dox). Our analysis revealed that ATACseq peaks were associated with a greater percentage of cell cycle genes relative to the non-cell cycle genes (background genes) (p < 0.001) (Fig. 1c and Supplemental Table 3). Based on these results, we hypothesized that Etv2 promoted cellular proliferation by regulation of cell cycle genes. To further examine whether Etv2-expressing cell populations were associated with cellular proliferation during embryogenesis, we used transgenic reporter mice (*Etv2*-*EYFP*) that expressed EYFP under the control of the 3.9 kb *Etv2* promoter^5^. Previously, we showed that the 3.9 kb *Etv2* promoter driving EYFP marks the earliest hemato-endothelial lineages during development^5^. We harvested E8.5 *Etv2*-*EYFP* transgenic embryos and performed immunohistochemical analysis using Ki67 (a marker for cell proliferation) and an antibody that recognizes the EYFP protein. This analysis revealed colocalization of EYFP and Ki67 in the blood-island regions of the yolk-sac in the developing embryo (Fig. 2a). Our quantitative analysis revealed that 22% ± 4% of the EYFP^+^ cells were colocalized with Ki67 in these embryos (Fig. 2b). To further investigate the connection between Etv2 expression and cell proliferation, we examined the expression of cell cycle transcripts using ESCs expressing zsGreen1-DR under the control of the 3.9 kb *Etv2* promoter^10,11^. We differentiated these ESCs into EBs and FACS-sorted for zsGreen^−^ and zsGreen^+^ cells at D3 and D4 and performed qPCR for transcripts that have a cell cycle function, including: *Ccnd2*, *Cdk7*, and *Cdkn2a*. The pro-proliferative cell cycle transcripts *Ccnd2* and *Cdk7* in zsGreen^+^ cells were significantly increased relative to zsGreen^−^ cells at both time points. In contrast, we found unaltered or decreased expression of the cell cycle repressor, *Cdkn2a*, in zsGreen^+^ cells vs. zsGreen^−^ cells at D3 and D4, respectively (n = 3; p < 0.01) (Fig. 2c–e). These results demonstrated that Etv2 expression was positively correlated with the expression of a pro-proliferative gene program.

### Etv2 promotes cellular proliferation during ES/EB differentiation

**Figure 3:**
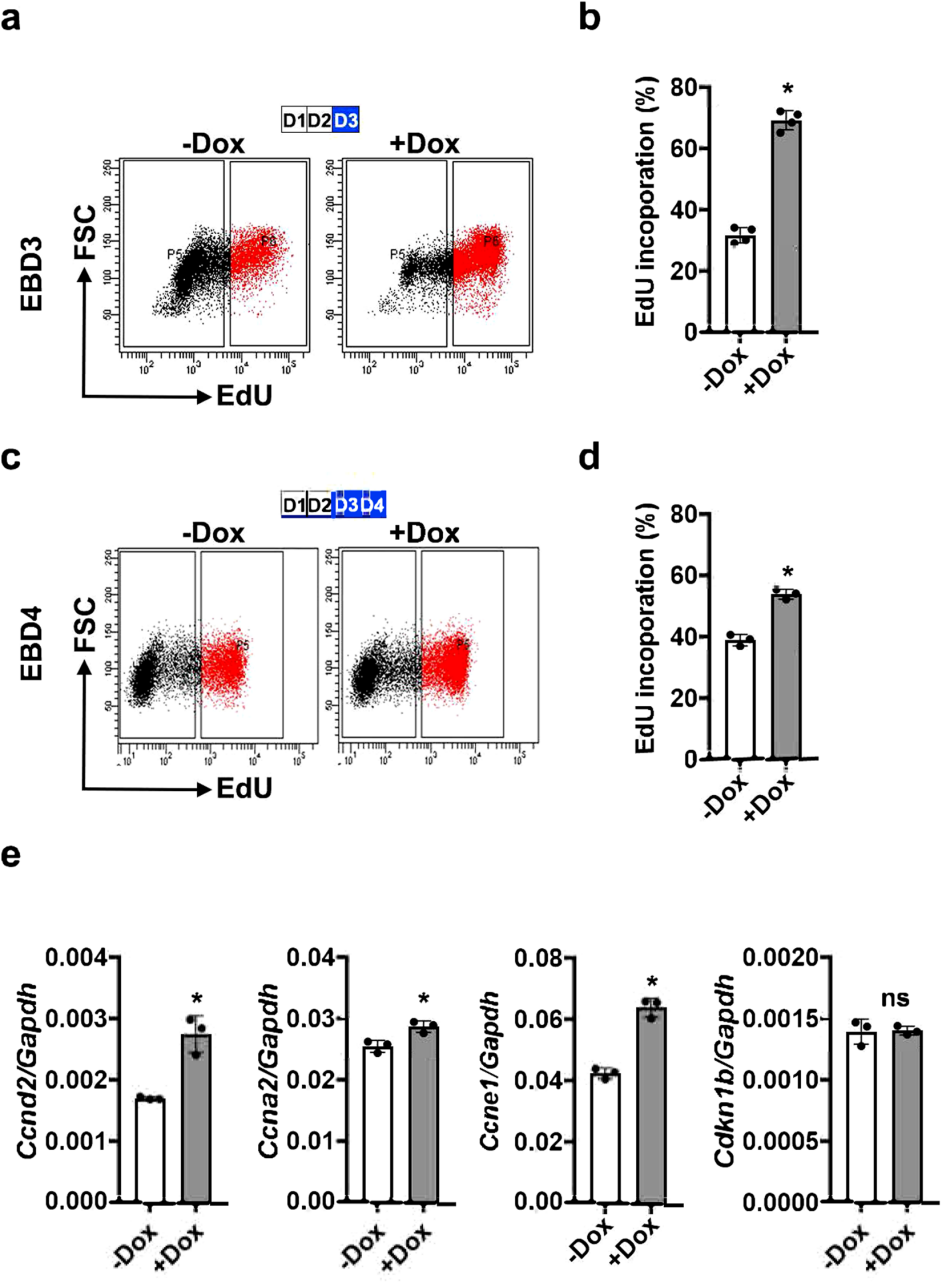
Induction of Etv2 promotes cellular proliferation in EBs. (**a**–**d**) FACS analysis (**a**,**c**) and quantification (**b**,**d**) of EdU-labelled cells from EBs in the absence (−Dox) and presence (+Dox) of Dox between D2-D3 and D2-D4. Dox induction of Etv2 resulted in significantly increased EdU labelling at both time points. Blue boxes indicate the timing of Dox treatment. (**e**) qPCR analysis of cell cycle gene expression in *iHA*-*Etv2* ES/EBs differentiated in the absence (−Dox) and presence (+Dox) of Dox. Pro-proliferative cell cycle genes *Ccnd2*,*Ccna2* and *Ccne1* show increased expression with induction of Etv2. Expression of *Cdkn1b*, a cell cycle repressor, was not affected by Etv2 induction. Data are presented as mean ± SEM (n = 3 replicates; *p < 0.05).

Having established the correlation of Etv2 expression with cell cycle gene expression, we directly tested whether the activation of Etv2 could induce cellular proliferation during ES/EB differentiation. During ES/EB differentiation, expression of Etv2 is transient, with the highest expression between D3 and D4 of differentiation^1,5,11^. To monitor the role of Etv2 as a promoter of cell proliferation, we differentiated *iHA*-*Etv2* ES/EB^8^ in the absence of Dox for 2 days and then treated EBs with Dox or vehicle (−Dox control) for an additional 24 h (D3) or 48 h (D4) and performed the EdU-labelling assay. The differentiating EBs were incubated with EdU (20 μM) for a 2 h period prior to harvest and analysis. Our EdU-incorporation assay revealed that induction of Etv2 resulted in a significant increase in the percentage of EdU-labelled cells at both D3 and D4 of differentiation in the induced EBs relative to uninduced EBs (n = 3; p < 0.01) (Fig. 3a–d and Supplemental Fig. 1a–d). To validate these results, we undertook qPCR analysis using RNA isolated from differentiating *iHA*-*Etv2* EBs in the presence or absence of Dox^8^. Consistent with the EdU labelling assays, our qPCR data showed that the levels of multiple cell cycle regulatory transcripts, including *Ccnd2*,*Ccna2* and *Ccne1*, were induced in Dox-induced EBs as compared to the uninduced EBs, while the levels of *Cdkn1b* were unchanged following Etv2 induction (n = 3; p < 0.01) (Fig. 3e). To further verify these findings, we performed western blot analysis for proliferating cell nuclear antigen (PCNA) using −Dox and + Dox cell lysate. Our data showed modest enrichment of PCNA in the + Dox condition relative to −Dox condition (Supplemental Fig. 2). Overall, these results indicated that the activation of Etv2 promoted cellular proliferation in EBs.

### Co-expression of *Yes1* in the Etv2^+^ cells

**Figure 4:**
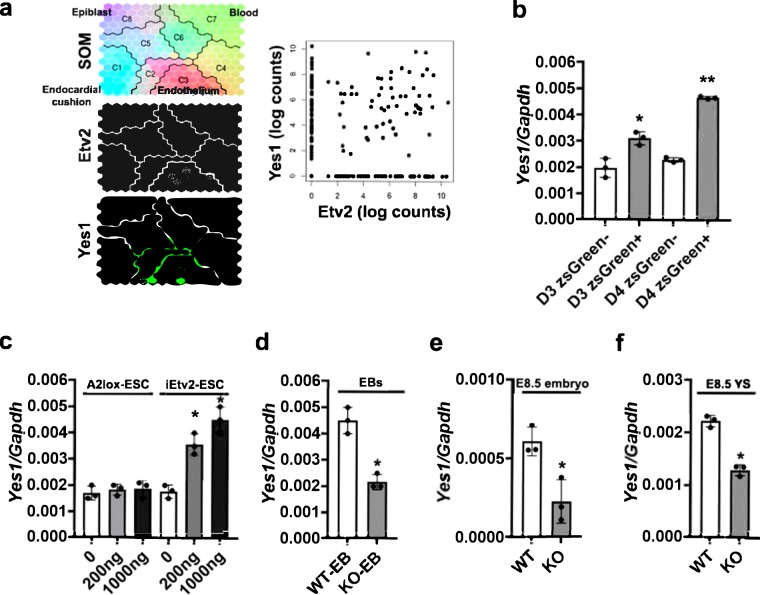
*Yes1* is co-expressed with *Etv2* during embryogenesis. (**a**) Left panel: Dpath analysis of single cell RNAseq data^10,27^ from *Etv2*-*EYFP* embryos^5^ at E7.25, E7.75, and E8.25. Note both *Yes1* and *Etv2* were highly expressed in the endothelial lineage. Right panel: Visualization of *Yes1* and *Etv2* expression within each cell population. Note that high Etv2 expression is positively correlated with high *Yes1* expression. (**b**) qPCR analysis for *Yes1* transcripts from zsGreen^−^ and zsGreen^+^ sorted cells using the *Etv2*- zsGreen1-DR ES/EB system^11^ at D3 and D4 of differentiation. Note a significant enrichment of *Yes1* in the zsGreen^+^ cells relative to the zsGreen^−^ cells. (**c**) qPCR analysis for *Yes1* transcripts from –Dox and increasing concentrations of Dox using RNA isolated from A2Lox and iHA-Etv2 ES/EB system^8^. Note a significant enrichment of *Yes1* in the induced EBs relative to A2Lox EBs. (**d**) qPCR analysis for *Yes1* transcripts from wildtype (WT) and *Etv2* null (KO) ES/EBs. The expression levels of *Yes1* were reduced in the *Etv2* null EBs relative to WT EBs. (**e**,**f**) qPCR analysis for *Yes1* transcripts from wildtype (WT) and *Etv2* null (KO) embryos and yolk sacs (YS) at E8.5. The levels of *Yes1* were reduced in the *Etv2* null embryos and *Etv2* null yolk sacs compared to controls. Data are presented as mean ± SEM (n = 3 replicates; **p < 0.01; *p < 0.05).

To decipher the role of Etv2 with cell cycle regulatory factors, we initially clustered our published bulk RNAseq datasets^10^ from the *iHA*-*Etv2* ES/EB system^8^ at 6 h and 12 h and compared the transcripts between −Dox and + Dox conditions. The transcript clustering strategy was based on three criteria: i) robust differential expression ( > 1.2 fold change) between + Dox and −Dox conditions; ii) significant differential expression (p < 0.01) following Dox treatment at the 6 h period; and iii) annotation using the GO-classification for cell cycle progression/differentiation. Based on these criteria, we identified *Yes1* as one of the top-ranked candidates involved in cellular proliferation following Dox treatment (Fig. 1b). The role of Yes1 as a regulator of cellular proliferation during development and tumor formation has been previously established^26^. To further examine whether *Etv2* and *Yes1* were co-expressed during embryogenesis, we analyzed the published single cell RNAseq (scRNAseq) datasets obtained from *Etv2*-*EYFP* transgenic embryos^5^ at E7.25, E7.75 and E8.25 using Dpath Software^27^. Our scRNAseq analysis showed robust overlap between *Etv2* and *Yes1* expression in the hematoendothelial lineage and limited expression in other lineages (Fig. 4a; left panel). Further, the expression analysis of *Yes1* and *Etv2* showed a positive correlation within the single cell population (Fig. 4a; right panel). To validate these results, we utilized the previously described *Etv2*-*zsGreen1*-*DR* ES/EB system^11^ and sorted zsGreen^−^ and zsGreen^+^ cells at D3 and D4 of differentiation and performed qPCR for *Yes1* transcripts. Our results indicated that *Yes1* was expressed in both zsGreen^−^ and zsGreen^+^ cell populations (Fig. 4b). Further, our analysis showed a modest but significant increase in *Yes1* expression in zsGreen^+^ cells compared to zsGreen^−^ cells at D3 of differentiation (n = 3; p < 0.01). The increase in *Yes1* expression in the zsGreen^+^ cells relative to zsGreen^−^ cells was more profound at D4 of differentiation (n = 3; p < 0.01). These results supported the hypothesis that *Etv2* and *Yes1* were co-expressed in hemato-endothelial lineages both *in vivo* and *in vitro* during embryogenesis. To monitor whether expression of *Yes1* was modulated by Etv2 during ES/EB differentiation, we utilized the A2lox ES/EB and *iHA*-*Etv2* ES/EB system^8^ and performed a dose response study by varying the concentrations of Dox. Then we isolated RNA from the EBs and performed qPCR from these EBs. Our qPCR analysis revealed that Dox-mediated induction of Etv2 resulted in increased levels of *Yes1* transcript in a dose dependent fashion (Fig. 4c). Next, we performed qPCR analysis using *Etv2* null ES/EBs and found significantly reduced levels of *Yes1* transcripts in *Etv2* null EBs as compared to wildtype EBs (Fig. 4d). To further validate these results, we performed qPCR analysis for *Yes1* transcripts using RNA isolated from wildtype and *Etv2* null embryos or yolk sacs (YS) at E8.5 (Fig. 4e,f)^2^. We found robust expression of *Yes1* in wildtype embryos but significantly lower expression in *Etv2* null embryos and yolk sacs (Fig. 4e,f). Together, these results indicated that *Etv2* and *Yes1* were co-expressed in the developing embryo.

### Etv2 is an upstream regulator of *Yes1* during embryogenesis

**Figure 5:**
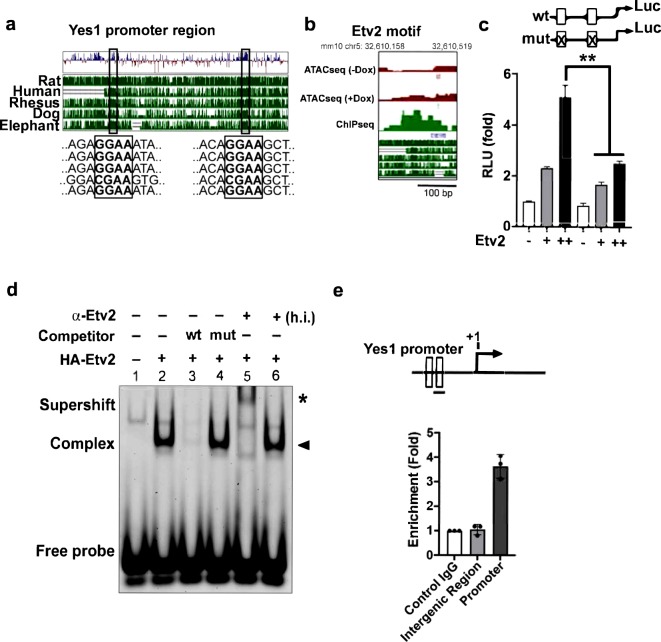
*Yes1* is a downstream target of *Etv2*. (**a**) Evolutionary conservation of the 5.0 kb upstream promoter fragment of the *Yes1* gene. Note the high conservation of the Etv2 binding motif across various species. (**b**) Alignment of ATACseq peak and ChIPseq peak within the *Yes1* upstream region. (**c**) Luciferase reporter constructs using the *Yes1* promoter (0.5 kb) harboring wildtype (wt; open box) or mutant (mut; crossed box) Etv2 binding sites. Etv2 enhanced the transcriptional activity in a dose-dependent manner. (**d**) EMSA showing Etv2 bound to the Ets binding site in the *Yes1* promoter region. IRdye-labeled probes containing the putative binding sites were incubated with *in vitro* synthesized HA-ETV2 protein to form a specific complex with the oligo (lane 2; arrowhead), which is competed with wildtype unlabeled oligos (lane 3) but not with mutant (lane 4). Addition of the HA-antibody supershifted the complex but not with heat-inactivated (h.i.) antibody (asterisk), indicating specificity of the complex. (**e**) Top: Schematic of the upstream region of the *Yes1* promoter showing the Etv2 binding sites (open boxes). Bottom: ChIP analysis of D4 Dox-inducible iHA-Etv2 EBs using an HA antibody. ChIP assay for the *Gapdh* promoter was used as a control. ChIP assay using an intergenic region was performed to validate the specificity. Data are presented as mean ± SEM (n = 3 replicates; **p < 0.01).

Based on these results, we hypothesized that Yes1 was a downstream effector of Etv2 in the regulation of cellular proliferation during differentiation. To monitor whether Etv2 regulated the expression of *Yes1*, we initially analyzed the upstream region of the *Yes1* locus and identified evolutionary conserved Etv2 binding motifs among various mammalian species (Fig. 5a and Supplemental Fig. 3). Next, we observed that the *Yes1* promoter region had an aligned Etv2 ChIPseq peak with an ATACseq peak following over-expression of Etv2 (Fig. 5b). To test whether Etv2 could bind to the upstream region of *Yes1* promoter and regulate expression of *Yes1*, we performed transcriptional assays using the 0.5 kb *Yes1* promoter that harbored the evolutionary conserved Etv2 binding motif fused to a luciferase reporter (n = 3; p < 0.01). Co-transfection of the *Yes1*-*promoter*-reporter plasmid with increasing amounts of *Etv2* expression plasmid resulted in a dose-dependent increase in luciferase activity (~5-fold) relative to co-transfection of the reporter with the empty expression plasmid (n = 3; p < 0.05). Mutations in the Etv2 binding motifs resulted in attenuated activation of the *Yes1*-*promoter*-*reporter* construct by Etv2 (n = 3; p < 0.05) (Fig. 5c). We next performed electrophoretic mobility gel shift assays (EMSAs) using double stranded DNA oligonucleotides (oligos) containing the evolutionary conserved Etv2 binding motif in the *Yes1* promoter. Incubation of *in vitro* synthesized Etv2 with an IRdye-labelled *Yes1* promoter oligo (probe) resulted in the formation of a protein-DNA complex, indicating the binding of Etv2 to this sequence (Fig. 5d and Supplemental Fig. 4). This binding of Etv2 was blocked by the addition of an unlabeled oligo (competitor) but not by the addition of a mutant competitor, indicating that Etv2 binding to these oligos was sequence specific. In addition, the protein-probe complex could be supershifted with an Etv2-specific antibody but could not be supershifted with a denatured (heat-inactivated; h.i.) anti-Etv2 antibody, indicating that the mobility shift seen was mediated by Etv2 (Fig. 5d and Supplemental Fig. 4). To determine whether Etv2 binds the *Yes1* promoter *in vivo*, we performed chromatin immunoprecipitation (ChIP) using Dox-induced cell lysates from the *iHA*-*Etv2* ES/EB system. Enrichment of the Etv2 binding was determined by qPCR experiments relative to *Gapdh* as control. Our analysis revealed ~4-fold enrichment of Etv2 in the *Yes1* promoter region relative to the *Gapdh* promoter (Fig. 5e). The binding of Etv2 to the *Yes1* promoter region was highly specific, as ChIP-qPCR for an intergenic region did not show any enrichment (Fig. 5e). These results indicated that Etv2 binds to the *Yes1* promoter and regulates its gene expression during embryogenesis.

## Discussion

Intersections between signaling pathways and transcriptional networks are important for an enhanced understanding of the mechanisms that govern development and disease^28–33^. These networks have been shown to govern cell fate of progenitors during embryogenesis^2,34,35^. For example, signaling pathways such as Notch, Vegf, Shh, etc. have been shown to interact with multiple factors including Hes1, Gli1, Etv2 and others to regulate hemato-endothelial development^8,11,36–38^. These networks interact extensively to facilitate the common mesodermal progenitors to expand and differentiate to give rise to multiple mesodermal derivatives^5,38^. However, the mechanisms that govern these networks and their interactions are incompletely defined. We and others have shown that *Etv2* mutants completely lack hemato-endothelial lineages and are lethal early during embryogenesis^2,5,6,8^. Recently, we have shown that Etv2 and signaling pathways including Pdgfra signaling, Flk1/Flt1 cascade, CREB signaling and hedgehog signaling play an important role in the modulation of hemato-endothelial lineages, highlighting the impact of these pathways on the Etv2 lineages^1,8,11^. In the present study, we utilized high throughput sequencing strategies, genetic mouse models and an inducible ES/EB model to overexpress Etv2 and have made three fundamental discoveries to decipher the mechanistic role of Etv2 in cellular proliferation during embryogenesis.

Our first discovery focused on the role of Etv2 as a regulator of cellular proliferation. Using ChIPseq databases, we demonstrated that Etv2 binds to the upstream regulatory regions of cell cycle regulatory genes. We further observed that the induction of Etv2 led to increased cellular proliferation in ESC/EBs. Recent studies have elucidated the essential role of endothelial progenitor cells (EPCs) in vascular regeneration^39^, however, the factors and molecular mechanisms that govern the EPCs are not completely defined. Identification of such mechanisms may serve as targets to promote lineages developmentally or cardiovascular regeneration following ischemic injury. In the present study, we defined Etv2 as a factor, which positively regulated cellular proliferation. The mechanisms whereby Etv2 regulated cell proliferation were the foundation for our second discovery.

Our second discovery defined Etv2 as a direct upstream regulator of *Yes1* gene expression. Previous studies have demonstrated the role of Yes1 as an activator of YAP activation thereby modulating the Hippo signaling pathway during development^22^. Recent studies have examined the role of Hippo signaling in cardiovascular development and regeneration^40,41^. Using gene disruption technology in the mouse, YAP (Yes Associated Protein) mutants resulted in embryonic lethality by E8.5 due to vascular defects and retarded growth^20^. Whether the lethality and growth retardation were due to defective proliferation is unclear. Similar to the global knockout, the *Nkx*-*2*.5-*Cre*-mediated deletion of *floxed*-*YAP* resulted in embryonic lethality due to defective cardiomyocyte proliferation and development^42^. Although a number of studies have documented the role of Hippo signaling, its mechanistic role and regulation have not been completely defined in the endothelial lineages.

Several studies have demonstrated the phosphorylation dependent modulation of YAP activity. Among the Src kinase family, only Yes1 has been shown to have a functional role in increased cell proliferation^43^. Our data provide a regulatory mechanism whereby the expression of Yes1 (a critical modulator of the Hippo signaling pathway) is activated during embryogenesis. In the present study, we showed that Etv2 binds to the upstream regions of the *Yes1* promoter and regulates its expression during embryogenesis. Using an array of techniques, we demonstrated that *Yes1* was a direct downstream target of Etv2. Our analysis using *Etv2* knockout embryos showed that the expression of *Yes1* was significantly reduced but not completely absent, suggesting that there might be additional regulators of *Yes1* (in other lineages) during embryogenesis. Previous studies have shown that Yes1 activation of YAP resulted in translocation to the nuclear compartment and formation of a β-catenin/Tbx5/YAP1 complex that promoted cellular proliferation^22^. Importantly, aberrant activation of Yes1 led to increased proliferation and tumor formation^22^. Moreover, since Etv2 has been shown to be expressed transiently during embryogenesis, we predict that elevated levels of Yes1 (Yes1^hi^) might be important to have a regulatory role for β-catenin dependent modulation within the hemato-endothelial lineages. In agreement with our prediction, we found multiple signaling pathways including Notch, FGF and Wnt signaling in the regulation of early developmental events in a context-dependent manner. For example, the modulation of FGF-Erk1/2 signaling resulted in the loss of Pdgfra^+^ cells^44,45^, whereas a distinct wave of Wnt signaling resulted in the regulation of cardiogenesis and hematopoiesis in a temporal fashion^46^. Previous studies have also shown the importance of Wnt signaling in hemato-endothelial development and Wnt signaling upstream of Etv2^6^. Moreover, the function of Wnt signaling has been well established in cell proliferation and regeneration^47,48^. Future studies will be necessary to define whether Etv2 induction promotes YAP phosphorylation and activation.

Our third discovery was the mechanism whereby Etv2 modulated the expression of cell cycle regulators. Our ChIPseq analysis showed Etv2 binding sites within a number of cell cycle genes and resulted in enrichment of multiple cell cycle transcripts including *Ccnd1*, *E2f4*, and *Ccne1*. Based on the ATACseq analysis, we proposed that the binding of Etv2 to the regulatory regions of the cell cycle genes resulted in higher chromatin accessibility following the induction of Etv2. The mechanism regarding the ability of Etv2 to open the closed chromatin structures and/or serve as an epigenetic modulator will be further examined in future studies.

In summary, we showed a novel role for Etv2 as an upstream regulator of *Yes1*, which modulates the Hippo signaling pathway and promotes cellular proliferation. Further, we defined a role for Etv2 in the promotion of chromatin accessibility. Collectively, these studies emphasize the important role for Etv2 as a regulator of cellular proliferation during embryogenesis.

## Methods

### Immunohistochemistry

All animal studies were approved by the Institutional Animal Care and Use Committee at the University of Minnesota. All methods were performed in accordance with the relevant guidelines and regulations. Time-mated pregnant females were used to harvest stage-specific embryos. E8.5 embryos were fixed for 1 hour at 4 °C in 4% paraformaldehyde (PFA), washed twice in PBS, equilibrated using a sucrose gradient, embedded in OCT compound (Sakura) and cryosectioned. Immunohistochemistry was performed on 10 µm cryosections using standard procedures^48,49^. Briefly, cryosections were permeabilized with 0.2% Triton X-100 for 10 min, washed in PBS twice and blocked with immunohistochemical diluent (10% normal donkey serum, 0.1% Triton X-100 in PBS, pH 7.3) at room temperature. Sections were incubated with primary antibodies that included chicken anti-GFP (1:500, Abcam, ab13970) and rabbit anti-Ki67 (1:200, Abcam, ab15580) overnight at 4 °C. Sections on slides were washed with 0.1% PBST and incubated with secondary antibodies that included anti-chicken Dylight 488 (1:400) and anti-rabbit Cy3 (1:400) (Jackson ImmunoResearch Laboratories) sera. Sections on slides were washed with 0.1% PBST, incubated with DAPI (1X) solution for 10 min at room temperature, washed with PBS twice and mounted using mounting media (Vectalabs). Immunostained sections were imaged on a Zeiss Axio Imager M1 upright microscope and processed using Adobe Photoshop CS6 software.

### RNA isolation and qPCR analysis

Total RNA was isolated from Etv2 wildtype and knockout embryos^2^, FACS-sorted cells or cells from EBs using the RNeasy kit (Qiagen) according to the manufacturer’s protocol. cDNA was synthesized using the SuperScript IV VILO kit (Thermo Fisher Scientific) according to the manufacturer’s protocol. Quantitative PCR (qPCR) was performed with ABI Taqman probe sets (Supplemental Table 4).

### Embryonic stem (ES) cell and embryoid body (EB) cultures

A2lox ESCs and doxycycline-dependent Etv2-overexpressing ESCs were generated using an inducible cassette exchange strategy as described previously^8^. In this system, iHA-Etv2 cells treated with 0.5 μg/ml doxycycline overexpressed Etv2 tagged with the HA epitope (*HA*-*Etv2*). To mimic early embryonic development, A2lox ESCs, iHA-Etv2 ESCs and Etv2-zsGreen-DR1 ESCs were differentiated into embryoid bodies (EBs) using mesodermal differentiation media as previously described^8^. Briefly, ESCs were dissociated into a single cell suspension using 0.25% trypsin and differentiated using the shaking method in differentiation media containing 15% FBS (Foundation ES Cell serum), 1X penicillin/streptomycin, 1X GlutaMAX (Gibco), 50 μg/ml Fe-saturated transferrin, 450 mM monothioglycerol, 50 μg/ml ascorbic acid in IMDM (Invitrogen). EBs were treated with doxycycline between day 2 and day 4 of differentiation, as specified for each experiment and harvested.

### EdU incorporation assay

Differentiating EBs derived from A2lox or iHA-Etv2 ESCs were incubated with 20 μM EdU for 2 hours. Treated EBs were then dissociated into a single-cell suspension with 0.25% trypsin. EdU staining of single cells was performed using the EdU labeling kit (Life Technologies) as per the manufacturer’s protocol. EdU labeled cells were FACS analyzed using a FACSAriaII as previously described^30^ and data were assembled using Adobe Photoshop CS6 software.

### Electrophoretic mobility shift assay

pcDNA3.1-Etv2-HA or empty pcDNA3.1(+)-HA vectors were expressed using the TNT Quick Coupled Transcription/Translation System (Promega, Madison, WI) according to the manufacturer’s protocol. DNA oligonucleotides (oligos) corresponding to wildtype *Yes1* promoter sequence (WT) or the sequence with an AGG (or complementary TTC) mutation in the putative Etv2 binding site (mutant) were synthesized with and without the IRDye® 700 fluorophore (Integrated DNA Technologies, Coralville, IA). *Yes1* WT top labeled: IRD700-TACAGTCAACAGGAAGCTTCTGCGG; *Yes1* WT top: TACAGTCAACAGGAAGCTTCTGCGG; *Yes1* WT bottom:CCGCAGAAGCTTCCTGTTGACTGTA; *Yes1* mutant top: TACAGTCAACTTCAAGCTTCTGCGG; *Yes1* mutant bottom: CCGCAGAAGCTTGAAGTTGACTGTA. Complimentary WT or mutant oligos were annealed to generate labeled probe and unlabeled competitor DNA. *In vitro* synthesized HA-Etv2 (1 µL) was pre-bound with 250 ng of poly dI-dC (Sigma) in binding buffer (50 mM Tris pH 7.6, 80 mM NaCl, 8% glycerol) at room temperature for 10 minutes. Pre-binding reactions included 5 nmol of unlabeled competitor oligo as appropriate. For supershift assays, pre-binding of Etv2 was performed in the presence of active or heat-inactivated anti-human Etv2 antibody (ER71 (N-15), catalog #sc-164278; Santa Cruz Biotechnology, Inc., Dallas, TX). IRDye® 700-labelled probe (100 fmol) was then added to the pre-binding reaction and then incubated at room temperature for 15 minutes. Protein-probe complexes were resolved on a 6% non-denaturing polyacrylamide gel in 0.5x TBE (40 mM Tris pH 8.3, 45 mM boric acid, and 1 mM EDTA) at room temperature. Fluorescence was detected using an Odyssey CLx imager (LI-COR Biosciences, Lincoln, NE).

### Bioinformatics analysis

We used a published Etv2 ChIPseq dataset^25^ and the R package GenomicRanges 1 (v1.30.3) to identify genes associated with Etv2 ChIP-seq peaks. Etv2 peak-associated genes were filtered for those genes annotated with the term cell cycle (GO:0007049) using R package GO.db 2 (v3.5.0). Significance values were determined using the Fisher Exact test. For ATACseq analysis of chromatin accessibility, 50,000 cells each from iHA-Etv2 ESCs or iHA-Etv2 ESC-derived EBs at day 2 or day 3 of differentiation in the presence of Dox were submitted to the Genomics Core (UMGC, UMN) in duplicate. The reads were mapped to the reference mouse genome (mm10) using Bowtie 3 (v2.2.2) and deduplicated using samtools 4 (v1.5). Peak calling was performed on the resulting bam files using the MACS2 (v2.1.1) function callpeak. GenomicRanges 1 (v1.30.3) was used to identify genes associated with ATACseq peaks. Significance values were determined using the Fisher Exact test. To identify genes regulated by Etv2, we re-analyzed our previously published bulk RNAseq data^10^ of the *iHA*-*Etv2* ES/EB system following 6 h and 12 h of Dox treatment. Differential gene analysis was performed using the R package, DESeq2 7 (v1.18.1), to obtain normalized counts, fold change, and p-values. Genes were considered significant if the p-value was less than 0.05 and absolute fold change was greater than 1.2. Normalized expression was log-transformed and scaled to generate heatmaps. Heatmaps were generated using the R package pheatmap 8 (v1.0.8).

### Luciferase assays

Luciferase reporter constructs (*Yes1*-*Luc*) were generated with luciferase (*Luc*) under the control of either a 0.5 kb fragment of the *Yes1* promoter harboring two evolutionarily conserved Etv2 binding motifs or the same fragment with inactivating mutations of the Etv2 binding motifs. The *Yes1* promoter region was amplified using PCR and subcloned into the pGL3 vector to generate pGL3-*Yes1*-*Luc*. Cos-7 cells were grown in Dulbecco’s modified Eagle’s complete medium supplemented with 10% FBS and 1X penicillin/streptomycin (ThermoFisher Scientific). Cos-7 cells were trypsinized using 0.25% trypsin and 1 × 10^5^ cells were plated in each well of a 12-well plate and co-transfected with wildtype (WT) or mutant (mut) pGL3-*Yes1*-*Luc* and increasing amounts of Etv2 expression plasmid using Lipofectamine 3000 (Life Technologies) as per manufacturer’s protocol. Cells were transfected with 10 ng of pRL-CMV (Promega) expressing Renilla luciferase as an internal control. Cos-7 cells were harvested 36 hours after transfection and luciferase activity quantified using the Dual Luciferase Stop-Glo System (Promega).

### Western blot analysis

Western blot analysis was performed as described previously^49^. Briefly, differentiating EBs following –Dox and + Dox treatment were lysed in ice-cold lysis buffer for 30 minutes and centrifuged at 10000 rpm for 10 min at 4 °C. Equal amounts of protein was loaded on 10% SDS-polyacrylamide gels. The PVDF membrane was blocked with 5% (w/v) milk protein and incubated with a goat-PCNA antibody [Santa Cruz, 1:1000] and LaminB1 antibody (Abcam; 1:1000) for an overnight period at 4 °C. The membrane was subsequently incubated with anti-goat HRP-conjugated secondary antibody and was visualized using SuperSignal West Femto Maximum Sensitivity Substrate kit (Thermo Scientific, USA) according to the manufacturer’s instructions. The protein bands were visualized and imaged using ImageLab 6.0.1 software.

### Chromatin Immunoprecipitation (ChIP)

*iHA*-*Etv2* ES/EBs were used for ChIP as previously described^8,50^. Briefly, EBs were disociated into single cells using 0.25% trypsin, fixed with 1% formaldehyde at room temperature for 10 min, and quenched in 0.125 M glycine. The cross-linked cell pellets (1–2 × 10^7^) were resuspended in lysis buffer (1% SDS, 5 mM EDTA, 50 mM Tris-HCl [pH 8.1], plus protease inhibitor) with gentle rocking at 4 °C for 10 min, followed by sonication to 200- to 500-bp fragments using an utrasonicator. The soluble lysate was diluted 10-fold in IP buffer (1% Triton X-100, 2 mM EDTA, 150 mM NaCl, 20 mM Tris-HCl [pH 8.1], 1 × protease inhibitor cocktail). Sonicated chromatin was precleared with protein G dynabeads and incubated with a HA-antibody (Sigma 12CA5) overnight at 4 °C. Subsequently, protein G- dynabeads were added and incubated for 2–3 h at 4 °C. The beads were then washed three times with cold wash buffer TSE I (1% Triton X-100, 150 mM NaCl, 0.1% SDS, 2 mM EDTA, 20 mM Tris-HCl, pH 8.1), TSE II (1% Triton X-100, 0.1% SDS, 20 mM Tris-HCl, pH 8.1, 2 mM EDTA, 500 mM NaCl), buffer III (0.25 M LiCl, 1% IGEPAL, 1% Deoxycholate, 1 mM EDTA, 10 mM Tris-HCl, pH 8.1) and then TE buffer. All buffers were supplemented with a protease inhibitor cocktail (Sigma P8340). Precipitated chromatin complexes were eluted in 100 μl elution buffer ((50 mM Tris 10 mM EDTA 1% SDS pH 8.0) at 65 °C for 10 minutes. The eluate were mixed with 10 µl 5 M NaCl for decrosslinking overnight at 65 °C, treated with RNase A for 2 h and then proteinase K for an additional 2 h period. DNA was purified with the PCR purification kit (Qiagen) and qPCR was performed using specific primers. The primers we used for our study were: Yes1 Promoter Fwd: 5′-CAC CAT TCC TGG GAG AAT-3′; Yes1 Promoter Rev: 5′-ACA CCT TGG TTC TCG TCT GG TG-3′; Intergenic control Fwd: 5′-TGG GCA TAT CCC TGG AGC TT-3′; Intergenic control Rev: 5′- GGC CAT CCC ACA GTC ACA AC-3′; Gapdh promoter Fwd: 5′- CATGGCCTTCCGTGTTCCTA-3′; Gapdh promoter Rev: 5-CTGGTCCTCAGTGTAGCCCAA-3′.

### Statistical analysis

All experiments were repeated at least three times and values presented are mean ± standard error of the mean (SEM). Statistical significance was determined using the Student’s *t*-test and a p-value ≤ 0.05 was considered as a significant change. For bioinformatics analysis, the significance was determined by using the Fisher Exact Test.

## Supplementary information

Etv2 transcriptionally regulates Yes1 and promotes cell proliferation during embryogenesis
