# Supplementary material for: Etv2 transcriptionally regulates Yes1 and promotes cell proliferation during embryogenesis
Source: Sci Rep. 2019 Jul 5;9:9736. doi: 10.1038/s41598-019-45841-5 (PMC6611806; doi:10.1038/s41598-019-45841-5)
Supplement: Supplementary file 1 — Etv2 transcriptionally regulates Yes1 and promotes cell proliferation during embryogenesis [file 41598_2019_45841_MOESM1_ESM.pdf]

Etv2 transcriptionally regulates *Yes1* and promotes cell proliferation during embryogenesis

Bhairab N. Singh[1], Wuming Gong[1], Satyabrata Das[1], Joshua W. M. Theisen[1,2], Javier E. Sierra-Pagan[1], Demetris Yannopoulos[1], Erik Skie[1], Pruthvi Shah[1], Mary G. Garry[1,3,4] and Daniel J. Garry[1,3,4]*

Supplemental Information

Supplemental Figure 1. Etv2 promotes EdU incorporation in the ES/EB system. (a, c) Pseudocolor representation of the FACS plots (related to Figure 3) shown here for improved resolution. (b, d) Quantification of EdU-labelled cells from EBs in the absence (-Dox) and presence (+Dox) of Dox between D2-D3 and D2-D4. Data are presented as mean $\pm$ SEM (n = 3 replicates; * $p < 0.05$).

Supplemental Figure 2. Etv2 promotes cellular proliferation. Western blot analysis of proliferating cell nuclear antigen (PCNA) using -Dox and +Dox cell lysates. Our data showed modest enrichment of PCNA in the +Dox condition relative to -Dox condition.

Supplemental Figure 3. *Yes1* is a downstream target of *Etv2*. Wider view of evolutionary conservation of the upstream promoter fragment of the *Yes1* gene. Note the high conservation of the Etv2 binding motif across various species. The box region indicates the Etv2 binding motif with the ChIPseq peak.

Supplemental Figure 4. *Yes1* is a downstream target of *Etv2*. The EMSA gel figure is the uncropped image used for Figure 5C. The dotted line indicates the cropped ESMA (right side) used to generate Figure 5C.

Supplemental Table 1. Etv2 ChIPseq peak analysis from the previously published datasets for the cell cycle genes.

Supplemental Table 2. Bulk RNAseq analysis from the previously published datasets for the cell cycle genes following following –Dox and +Dox treatment during ES/EB differentiation.

Supplemental Table 3. ATACseq analysis for the cell cycle genes following +Dox treatment during ES/EB differentiation.

Supplemental Table 4. List of qPCR taqman probes used in this study.

Figure S1 (Garry)

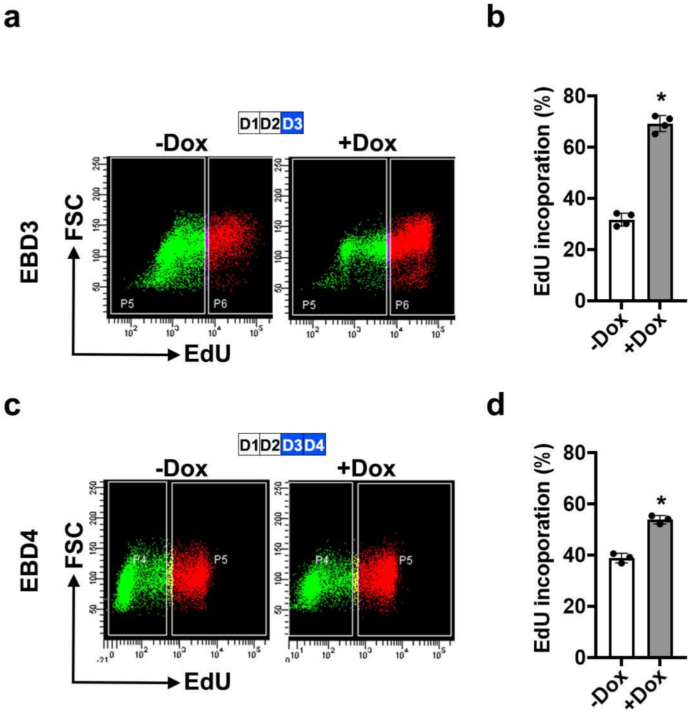

Figure S2 (Garry)

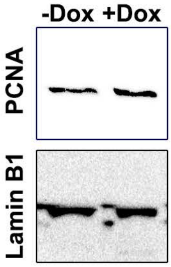

Figure S3 (Garry)

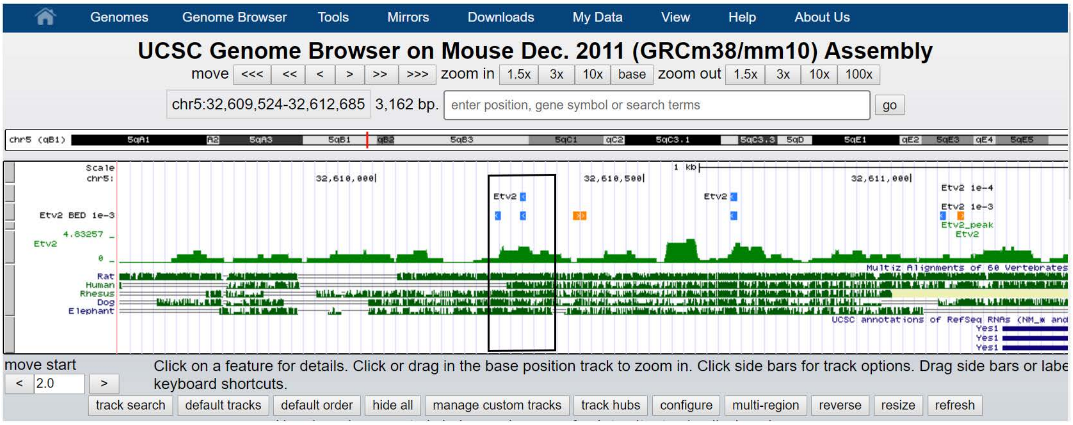

Figure S4 (Garry)

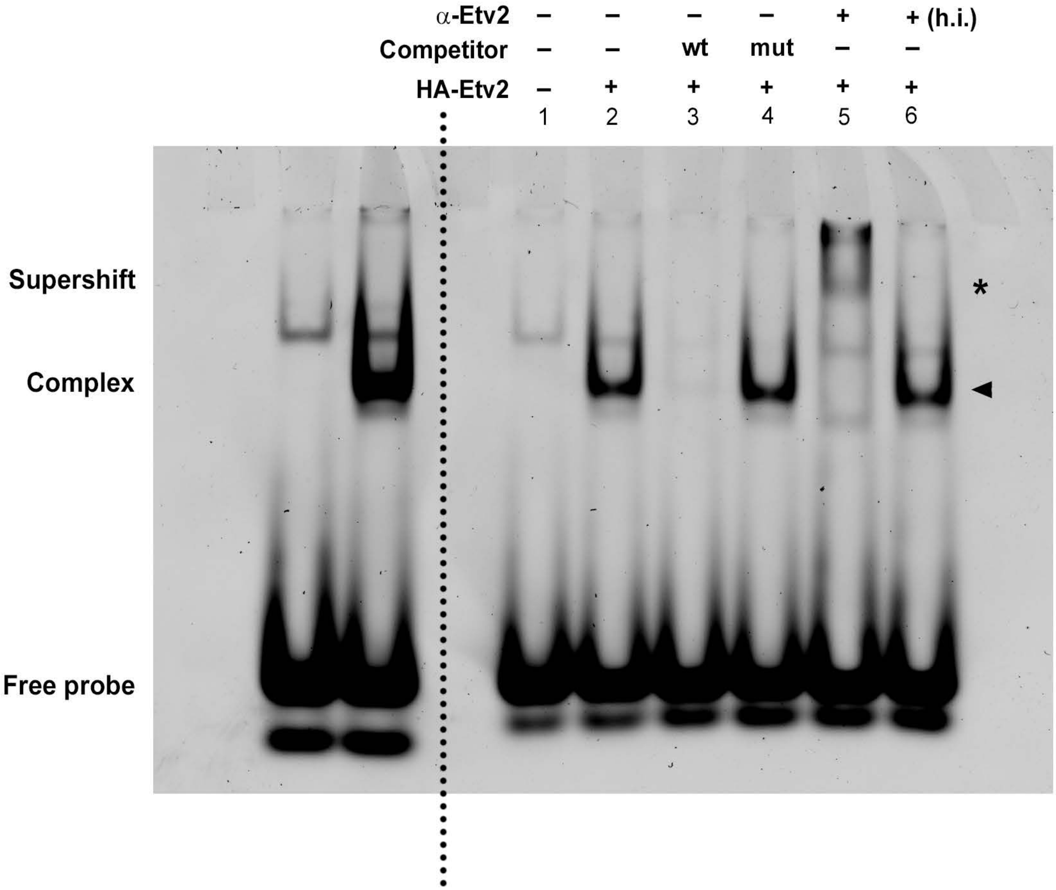

Supplemental Table 1. Etv2 ChIPseq peak analysis from the previously published datasets for the cell cycle genes.

SL	Gene Symbol	Chromosomes	From	To	Nearest Ensembl Gene ID	Distance From TSS
1	Cav2	chr6	17286505	17286605	ENSMUSG00000000058	-5370
2	Cav2	chr6	17286418	17286685	ENSMUSG00000000058	-5367
3	Nudt16	chr9	1.05E+08	105136752	ENSMUSG00000032565	-5317
4	Nudt16	chr9	1.05E+08	105136692	ENSMUSG00000032565	-5303
5	Hmcn1	chr1	1.51E+08	150997870	ENSMUSG00000066842	-4765
6	Hmcn1	chr1	1.51E+08	150997887	ENSMUSG00000066842	-4712
7	Git1	chr11	77497471	77497609	ENSMUSG00000011877	-3978
8	Git1	chr11	77497332	77497619	ENSMUSG00000011877	-3914
9	Fsd1	chr17	55990251	55990340	ENSMUSG00000011589	-3785
10	Yes1	chr5	32608089	32608295	ENSMUSG00000014932	-2979
11	Gdpd5	chr7	99384123	99384339	ENSMUSG00000035314	-2817
12	Zfp36l1	chr12	80115678	80115922	ENSMUSG00000021127	-2787
13	Dgkz	chr2	91968017	91968313	ENSMUSG00000040479	-2565
14	Junb	chr8	84978711	84978868	ENSMUSG00000052837	-2248
15	Junb	chr8	84978628	84978907	ENSMUSG00000052837	-2226
16	Tal1	chr4	1.15E+08	115060061	ENSMUSG00000028717	-2159
17	Junb	chr8	84978538	84978639	ENSMUSG00000052837	-2047
18	Clic1	chr17	35051753	35051924	ENSMUSG00000007041	-1873
19	Flt3l	chr7	45137426	45137764	ENSMUSG00000110206	-1508
20	Zfp36l1	chr12	80114265	80114531	ENSMUSG00000021127	-1385
21	Lrp5	chr19	3687686	3687779	ENSMUSG00000024913	-1177
22	Yes1	chr5	32610148	32610512	ENSMUSG00000014932	-1169
23	Trp53	chr11	69581403	69581628	ENSMUSG00000059552	-1157
24	Trp53	chr11	69581180	69581598	ENSMUSG00000059552	-1030
25	Hhex	chr19	37435636	37435963	ENSMUSG00000024986	-990
26	Uhrf1	chr17	56304143	56304309	ENSMUSG00000001228	-905
27	Parp3	chr9	1.06E+08	106477190	ENSMUSG00000023249	-899
28	Parp3	chr9	1.06E+08	106477108	ENSMUSG00000023249	-876
29	Dusp3	chr11	1.02E+08	101984317	ENSMUSG00000003518	-674
30	Ube2s	chr7	4812859	4813040	ENSMUSG00000060860	-599
31	Psmd13	chr7	1.41E+08	140882544	ENSMUSG00000025487	-451
32	Uvrag	chr7	99141502	99141634	ENSMUSG00000035354	-427
33	Uvrag	chr7	99141412	99141586	ENSMUSG00000035354	-358
34	Chmp3	chr6	71544060	71544212	ENSMUSG00000053119	-339
35	Crlf3	chr11	80081137	80081353	ENSMUSG00000017561	-307

36	Rnf167	chr11	70647430	70647593	ENSMUSG00000040746	-277
37	Rnf167	chr11	70647452	70647553	ENSMUSG00000040746	-268
38	Anapc13	chr9	1.03E+08	102626595	ENSMUSG00000035048	-262
39	Bag6	chr17	35135314	35135564	ENSMUSG00000024392	-261
40	Sdccag8	chr1	1.77E+08	176814984	ENSMUSG00000026504	-232
41	Actr3	chr1	1.25E+08	125435852	ENSMUSG00000026341	-201
42	Cdk20	chr13	64432460	64432530	ENSMUSG00000021483	-181
43	Cdk20	chr13	64432336	64432600	ENSMUSG00000021483	-154
44	Fignl1	chr11	11809035	11809184	ENSMUSG00000035455	-151
45	Aurka	chr2	1.72E+08	172370727	ENSMUSG00000027496	-147
46	Ppm1g	chr5	31220616	31220772	ENSMUSG00000029147	-129
47	Haus6	chr4	86612062	86612273	ENSMUSG00000038047	-113
48	Chmp5	chr4	40948456	40948552	ENSMUSG00000028419	-97
49	Prpf19	chr19	10895261	10895387	ENSMUSG00000024735	-93
50	Calm3	chr7	16924126	16924279	ENSMUSG00000019370	-89
51	Ccnl1	chr3	65958198	65958300	ENSMUSG00000027829	-87
52	Terb1	chr8	1.05E+08	104510007	ENSMUSG00000052616	-82
53	Ppm1g	chr5	31220494	31220746	ENSMUSG00000029147	-55
54	Champ1	chr8	13869594	13869796	ENSMUSG00000047710	-54
55	Prpf19	chr19	10895113	10895456	ENSMUSG00000024735	-54
56	Syf2	chr4	1.35E+08	134931057	ENSMUSG00000028821	-52
57	Chmp2a	chr7	13034555	13034851	ENSMUSG00000033916	-52
58	Babam1	chr8	71396836	71396986	ENSMUSG00000031820	-50
59	Wasl	chr6	24664967	24665146	ENSMUSG00000029684	-48
60	Washc1	chr17	66111525	66111658	ENSMUSG00000024101	-46
61	Cdk7	chr13	1.01E+08	100731079	ENSMUSG00000069089	-39
62	Appl2	chr10	83648652	83648891	ENSMUSG00000020263	-34
63	Chordc1	chr9	18292033	18292278	ENSMUSG00000001774	-31
64	Mad2l1	chr6	66535329	66535511	ENSMUSG00000029910	-30
65	Gnai3	chr3	1.08E+08	108146249	ENSMUSG00000000001	-25
66	Cdk7	chr13	1.01E+08	100731100	ENSMUSG00000069089	-25
67	Stk4	chr2	1.64E+08	164074290	ENSMUSG00000018209	-21
68	Ccp110	chr7	1.19E+08	118712655	ENSMUSG00000033904	-21
69	Adam17	chr12	21373478	21373745	ENSMUSG00000052593	-19
70	Zbtb17	chr4	1.41E+08	141444750	ENSMUSG00000006215	-17
71	Chtf8	chr8	1.07E+08	106893646	ENSMUSG00000046691	-17
72	E2f4	chr8	1.05E+08	105297708	ENSMUSG00000014859	-10
73	Nup214	chr2	31974335	31974548	ENSMUSG00000001855	-6
74	Fam32a	chr8	72219648	72219822	ENSMUSG00000003039	-5

75	Kif3b	chr2	1.53E+08	153291486	ENSMUSG00000027475	-4
76	Stk4	chr2	1.64E+08	164074235	ENSMUSG00000018209	-2
77	Ccar2	chr14	70153659	70153967	ENSMUSG00000033712	-2
78	Itgb1	chr8	1.29E+08	128685708	ENSMUSG00000025809	+2
79	Ercc3	chr18	32240211	32240375	ENSMUSG00000024382	+7
80	Thoc5	chr11	4895275	4895348	ENSMUSG00000034274	+9
81	Cltc	chr11	86757449	86757650	ENSMUSG00000047126	+16
82	Bbs4	chr9	59353416	59353569	ENSMUSG00000025235	+16
83	Fam32a	chr8	72219608	72219817	ENSMUSG00000003039	+18
84	Thoc5	chr11	4895142	4895454	ENSMUSG00000034274	+22
85	Txnip	chr3	96557837	96558033	ENSMUSG00000038393	+22
86	Itgb1	chr8	1.29E+08	128685725	ENSMUSG00000025809	+23
87	Mad2l1	chr6	66535209	66535522	ENSMUSG00000029910	+25
88	Phactr4	chr4	1.32E+08	132422500	ENSMUSG00000066043	+26
89	Ing4	chr6	1.25E+08	125039842	ENSMUSG00000030330	+30
90	Ercc3	chr18	32240115	32240423	ENSMUSG00000024382	+31
91	Rps6	chr4	86857334	86857427	ENSMUSG00000028495	+32
92	Dapk3	chr10	81182880	81183065	ENSMUSG00000034974	+33
93	Khdrbs1	chr4	1.3E+08	129742342	ENSMUSG00000028790	+35
94	Ddx39b	chr17	35241561	35241861	ENSMUSG00000019432	+35
95	Cltc	chr11	86757447	86757603	ENSMUSG00000047126	+40
96	Pggt1b	chr18	46280771	46280841	ENSMUSG00000024477	+44
97	Gtpbp4	chr13	8995939	8996137	ENSMUSG00000021149	+45
98	Rps6	chr4	86857201	86857532	ENSMUSG00000028495	+46
99	Thap1	chr8	26158026	26158162	ENSMUSG00000037214	+47
100	Thap1	chr8	26158015	26158169	ENSMUSG00000037214	+49
101	Adam17	chr12	21373358	21373730	ENSMUSG00000052593	+49
102	Tbrg1	chr9	37657207	37657278	ENSMUSG00000011114	+70
103	Tbrg1	chr9	37657140	37657335	ENSMUSG00000011114	+75
104	E2f4	chr8	1.05E+08	105297762	ENSMUSG00000014859	+77
105	Rps3	chr7	99483577	99483743	ENSMUSG00000030744	+78
106	Cdk5rap3	chr11	96916337	96916486	ENSMUSG00000018669	+85
107	Ccar2	chr14	70153561	70153869	ENSMUSG00000033712	+96
108	C2cd3	chr7	1E+08	100372182	ENSMUSG00000047248	+126
109	Usp8	chr2	1.27E+08	126707335	ENSMUSG00000027363	+136
110	Rack1	chr11	48799983	48800405	ENSMUSG00000020372	+138
111	C2cd3	chr7	1E+08	100372186	ENSMUSG00000047248	+159
112	Rad51	chr2	1.19E+08	119112783	ENSMUSG00000027323	+164
113	Mnt	chr11	74830679	74830820	ENSMUSG00000000282	+171

114	Mnt	chr11	74830626	74830869	ENSMUSG00000000282	+173
115	Slbp	chr5	33652092	33652211	ENSMUSG00000004642	+194
116	Bax	chr7	45466460	45466706	ENSMUSG00000003873	+253
117	Fbxw11	chr11	32642364	32642539	ENSMUSG00000020271	+273
118	Casp8ap2	chr4	32615057	32615295	ENSMUSG00000028282	+302
119	Pin1	chr9	20651680	20651865	ENSMUSG00000032171	+323
120	Map4k2	chr19	6340730	6340892	ENSMUSG00000024948	+324
121	Arf1	chr11	59227857	59227981	ENSMUSG00000048076	+351
122	Cdk12	chr11	98202518	98202667	ENSMUSG00000003119	+467
123	Actr2	chr11	20112364	20112495	ENSMUSG00000020152	+484
124	Actr2	chr11	20112304	20112536	ENSMUSG00000020152	+493
125	Grk5	chr19	60889109	60889392	ENSMUSG00000003228	+499
126	Cdt1	chr8	1.23E+08	122567484	ENSMUSG00000006585	+589
127	Rps6ka2	chr17	7169275	7169552	ENSMUSG00000023809	+702
128	Ccdc124	chr8	70873076	70873363	ENSMUSG00000007721	+716
129	Lfng	chr5	1.41E+08	140606582	ENSMUSG00000029570	+807
130	Tal1	chr4	1.15E+08	115057020	ENSMUSG00000028717	+876
131	Vps4a	chr8	1.07E+08	107030441	ENSMUSG00000031913	+884
132	Mcm7	chr5	1.38E+08	138171328	ENSMUSG00000029730	+1169
133	Cdk1	chr10	69351258	69351507	ENSMUSG00000019942	+1528
134	Usp16	chr16	87452665	87453014	ENSMUSG00000025616	+1864
135	Arhgef2	chr3	88603957	88604236	ENSMUSG00000028059	+1870
136	Hmcn1	chr1	1.51E+08	150990815	ENSMUSG00000066842	+2351
137	Rassf2	chr2	1.32E+08	132027660	ENSMUSG00000027339	+2443
138	Mcm3	chr1	20817258	20817668	ENSMUSG00000041859	+2849
139	Pea15a	chr1	1.72E+08	172203935	ENSMUSG00000013698	+3015
140	Ing1	chr8	11555319	11555496	ENSMUSG00000045969	+3022
141	Bmp2	chr2	1.34E+08	133548849	ENSMUSG00000027358	+3390
142	Chd4	chr6	1.25E+08	125092646	ENSMUSG00000063870	+3439
143	Myh9	chr15	77838630	77838700	ENSMUSG00000022443	+3510
144	Gadd45g	chr13	51842827	51843159	ENSMUSG00000021453	+3685
145	Gadd45g	chr13	51842854	51843064	ENSMUSG00000021453	+3719
146	Csnk1a1	chr18	61550442	61550621	ENSMUSG00000024576	+4743
147	Myh9	chr15	77837205	77837399	ENSMUSG00000022443	+4873
148	Tal1	chr4	1.15E+08	115052963	ENSMUSG00000028717	+4998
149	Tal1	chr4	1.15E+08	115052970	ENSMUSG00000028717	+5034

Supplemental Table 2. Bulk RNAseq analysis from the previously published datasets for the cell cycle genes with Etv2 ChIPseq peak following –Dox and +Dox treatment during ES/EB differentiation.

SL	Symbol	Reference number	-Dox (6h)	+Dox (6h)	Relative exp.	-Dox (12h)	+Dox (12h)	Relative exp.
1	ETV2	NM_007959.1	706	10041	14.222	1440.3	8622.4	5.9865
2	CAV2	NM_016900.1	2	26.9	13.45	9	14	1.5556
3	TAL1	NM_011527.1	481.6	3885.9	8.0687	1020.8	5912.1	5.7916
4	LMO2	NM_008505.1	378	2515	6.6534	544	4033	7.4136
5	RASSF2	NM_175445.1	406.3	1466.4	3.6092	436.8	1408.1	3.2237
6	GADD45G	NM_011817.1	175	631	3.6057	188	910	4.8404
7	GATA2	NM_008090.1	411	1100	2.6764	855	3942	4.6105
8	HMCN1	NM_001024720.1	138	365	2.6449	257	573	2.2296
9	MAP4K2	NM_009006.1	651.1	1660.8	2.5508	711.2	1440	2.0247
10	HDAC7	NM_019572.1	1281	3011.9	2.3512	1858	3733.2	2.0093
11	YES1	NM_009535.1	1212.9	2812.6	2.3189	1414.4	2692.4	1.9036
12	E2F4	NM_148952.1	11676	20133	1.7243	11452	15813	1.3808
13	PEA15A	NM_011063.1	2115.3	3628.1	1.7152	2989	3507	1.1733
14	APPL2	NM_145220.1	524	869	1.6584	802.8	952	1.1858
15	GRK5	NM_018869.1	249	409	1.6426	267	388	1.4532
16	RPS6KA2	NM_011299.1	2728	4453	1.6323	3226	4384	1.359
17	FIGNL1	NM_021891.1	1251	1953	1.5612	1526	1922	1.2595
18	LFNG	NM_008494.1	188	291	1.5479	508	252	0.4961
19	TXNIP	NM_023719.1	2743	4134	1.5071	3395	3297.8	0.9714
20	ACTR2	NM_146243.1	5214.1	7667.9	1.4706	6042	7115	1.1776
21	HHEX	NM_008245.1	542.3	789.3	1.4555	799	1923.4	2.4073
22	BAX	NM_007527.1	4122	5950.3	1.4435	4565.3	6026	1.32
23	DUSP3	NM_028207.1	691.1	972.9	1.4078	752.8	1096.9	1.4571
24	UVRAG	NM_178635.1	741.8	1022.8	1.3788	729.6	1120.9	1.5363
25	MAD2L1	NM_019499.1	3601	4962	1.378	4367	4968	1.1376
26	FLT3L	NM_013520.1	28	38	1.3571	31	25	0.8065
27	SLBP	NM_009193.1	3712.7	5025.3	1.3535	4402.4	4804	1.0912
28	FBXW11	NM_134015.1	2735.4	3684	1.3468	3294	3528.1	1.0711
29	CASP8AP2	NM_011997.1	2435.2	3251	1.335	3055	2757	0.9025
30	GIT1	NM_001004144.1	5789	7671.4	1.3252	6831.8	7747.5	1.134
31	ERCC3	NM_133658.1	2080	2747	1.3207	2432	2565	1.0547
32	CRLF3	NM_018776.1	1455	1906	1.31	1631	2134	1.3084

33	UHRF1	NM_010931.1	15912	20626	1.2962	18627	18621	0.9997
34	TRP53	NM_011640.1	13665	17679	1.2937	15049	16093	1.0693
35	THOC5	NM_172438.1	2341	3022	1.2909	2851	2916	1.0228
36	MCM3	NM_008563.1	14779	18965	1.2832	16490	17985	1.0907
37	CDK12	NM_026952.1	5447.7	6975.6	1.2805	6268	6339.2	1.0114
38	ACTR3	NM_023735.1	5679.7	7262.7	1.2787	6946	7318.8	1.0537
39	CDK1	NM_007659.1	4170	5322	1.2763	5251.7	5315	1.0121
40	LRP5	NM_008513.1	6468.1	8113.2	1.2543	7415	8157.6	1.1001
41	MYH9	NM_022410.1	25400	31752	1.2501	28081	27458	0.9778
42	CDT1	NM_026014.1	4459.4	5544.2	1.2433	4898.3	4780	0.9758
43	DGKZ	NM_138306.1	4106.3	5064.1	1.2333	5405.3	5704.6	1.0554
44	THAP1	NM_199042.1	431	530	1.2297	542	651.3	1.2017
45	FSD1	NM_183178.1	647	793	1.2257	647	925	1.4297
46	CHMP5	NM_029814.1	1242	1520	1.2238	1490.8	1848	1.2396
47	ZBTB17	NM_009541.1	1105.7	1348	1.2191	1389	1468.8	1.0575
48	CSNK1A1	NM_146087.1	5946.6	7200	1.2108	6907	7307.8	1.058
49	BBS4	NM_175325.1	387.1	467	1.2064	576	409	0.7101
50	USP8	NM_019729.1	3661	4411.8	1.2051	4370.8	4052.5	0.9272
51	ING1	NM_011919.1	2044	2448	1.1977	2445	2320	0.9489
52	AURKA	NM_011497.1	4648.7	5548.2	1.1935	5244.6	5709.6	1.0887
53	CLTC	NM_001003908.1	12532	14864	1.1861	14549	12820	0.8812
54	NUP214	NM_172268.1	5529.9	6547.5	1.184	5976	5113.9	0.8557
55	MCM7	NM_008568.1	17672	20919	1.1838	19795	19094	0.9646
56	STK40	NM_028800.1	2507.5	2951	1.1769	3023	2418.1	0.7999
57	RPS6	NM_009096.1	59461	69930	1.1761	67757	68213	1.0067
58	FAM32A	NM_026455.1	3347.2	3935.5	1.1758	3692.2	3935.1	1.0658
59	CTCF	NM_181322.1	6519	7639	1.1718	7627	7411	0.9717
60	CDK7	NM_009874.1	2261.4	2648.1	1.171	2832	2572.4	0.9083
61	CCDC124	NM_026964.1	2627.7	3069	1.1679	2949.1	2713	0.9199
62	CHMP2A	NM_026885.1	2109.4	2463	1.1676	2310	2821	1.2212
63	CHD4	NM_145979.1	29638	34592	1.1672	31696	28519	0.8998
64	GTPBP4	NM_027000.1	5537	6460.5	1.1668	5950.6	6087.4	1.023
65	CHORDC1	NM_025844.1	2972.8	3447.3	1.1596	3378.5	3427	1.0144
66	C2CD3	NM_001017985.1	2564.6	2972	1.1589	3087.6	2704	0.8758
67	RAD51	NM_011234.1	2391.5	2766.8	1.1569	2835.1	2812.6	0.9921
68	RPS3	NM_012052.1	46395	53395	1.1509	52046	54206	1.0415
69	ARF1	NM_007476.1	12111	13897	1.1474	14242	13510	0.9486
70	CCNL1	NM_019937.1	1717	1968.6	1.1465	2001.9	1715.9	0.8571

71	PPM1G	NM_008014.1	10846	12414	1.1446	12042	11565	0.9604
72	MNT	NM_010813.1	2209.4	2526.3	1.1434	2524	2271	0.8998
73	WASL	NM_028459.1	3325.7	3742	1.1252	3874.8	3611.4	0.932
74	ARHGEF2	NM_008487.1	3923.5	4408.4	1.1236	4758.6	4310.9	0.9059
75	CALM3	NM_007590.1	13158	14755	1.1214	14786	16534	1.1182
76	UBE2S	NM_133777.1	14353	16068	1.1195	15329	16430	1.0718
77	ADAM17	NM_009615.1	1838.5	2044.7	1.1122	2282.4	2266.1	0.9929
78	PRPF19	NM_134129.1	12460	13792	1.1069	14295	12990	0.9088
79	SDCCAG8	NM_029756.1	151	166.9	1.1053	237.5	168	0.7074
80	CDK5RAP3	NM_030248.1	1840.4	2008.6	1.0914	2186.8	2114	0.9667
81	KIF3B	NM_008444.1	1089	1186	1.0891	1437.6	1302	0.9057
82	PSMD13	NM_011875.1	3355.4	3628.3	1.0813	3666	3610	0.9847
83	RNF167	NM_027445.1	1918.4	2066.6	1.0773	2270.8	2043.6	0.8999
84	TBRG1	NM_025289.1	3995	4255	1.0651	4508	3942	0.8744
85	ANAPC13	NM_181394.1	1218	1291	1.0599	1346	1369	1.0171
86	SYF2	NM_026780.1	1437	1519	1.0571	1718	1714.1	0.9977
87	DDX39B	NM_019693.1	14721	15511	1.0536	18128	14845	0.8189
88	CLIC1	NM_033444.1	3897.5	4083.8	1.0478	4864	5688.2	1.1694
89	ING4	NM_133345.1	716.8	746	1.0407	1039	891	0.8576
90	ZFP36L1	NM_007564.1	3435.8	3557	1.0353	4446.8	3736	0.8402
91	NUDT16	NM_029385.1	65	67	1.0308	80	99	1.2375
92	DAPK3	NM_007828.1	1712	1750.6	1.0225	1958	1729	0.883
93	JUNB	NM_008416.1	918	926	1.0087	1322	937	0.7088
94	ITGB1	NM_010578.1	7509.8	7520.2	1.0014	9651.8	7885.2	0.817
95	CHTF8	NM_145412.1	6984.1	6961	0.9967	8664.1	6893	0.7956
96	GDPD5	NM_201352.1	301	299	0.9934	402	511.9	1.2734
97	DNMT3B	NM_010068.1	55289	54700	0.9894	55988	48226	0.8614
98	GNAI3	NM_010306.1	4729	4620	0.977	5752	4396	0.7643
99	BMP2	NM_007553.1	307	294	0.9577	574	418	0.7282
100	VPS4A	NM_126165.1	938	847	0.903	1240	1020	0.8226
101	KHDRBS1	NM_011317.1	11060	9957.8	0.9003	12896	10603	0.8222
102	CDK20	NM_053180.1	223	183	0.8206	231	247	1.0693
103	HAND2	NM_010402.1	276	85	0.308	567	202	0.3563

Supplemental Table 3. ATACseq peak analysis for the cell cycle genes following +Dox treatment during ES/EB differentiation.

Gene symbol	Nearest Ensembl Id	log2FoldChange (ATACseq EBD3)	p-value
Mcm3	ENSMUSG00000041859	1.808834932	0.005517199
Hmcn1	ENSMUSG00000066842	1.502810045	0.021793135
Dgkz	ENSMUSG00000040479	1.095718937	0.057115498
Etv2	ENSMUSG00000006311	1.116360697	0.058115765
Tal1	ENSMUSG00000028717	1.208087781	0.066762917
Cav2	ENSMUSG00000000058	1.17175853	0.093669816
Pea15a	ENSMUSG00000013698	0.748359211	0.094698492
Vps4a	ENSMUSG00000031913	0.999169722	0.159667998
Rassf2	ENSMUSG00000027339	0.937929985	0.197220196
Hhex	ENSMUSG00000024986	0.924976829	0.205138959
Yes1	ENSMUSG00000014932	0.748517436	0.211689506
Myh9	ENSMUSG00000022443	0.746362933	0.276617861
Thoc5	ENSMUSG00000034274	0.45493221	0.288905019
Zbtb17	ENSMUSG00000006215	0.781508528	0.29121029
Hdac7	ENSMUSG00000022475	0.506977996	0.319535055
Gdpd5	ENSMUSG00000035314	0.423798435	0.342190459
Tal1	ENSMUSG00000028717	0.25461797	0.379128598
Fsd1	ENSMUSG00000011589	0.532667579	0.400058492
Tal1	ENSMUSG00000028717	0.236571314	0.410538455
Zfp36l1	ENSMUSG00000021127	0.604935017	0.414476101
Thoc5	ENSMUSG00000034274	0.370404495	0.423353136
Psmd13	ENSMUSG00000025487	0.265770688	0.446178577
Flt3l	ENSMUSG00000110206	0.432264445	0.46670459
Bmp2	ENSMUSG00000027358	0.411235783	0.553348073
Chd4	ENSMUSG00000063870	0.428212692	0.568262243
Lfng	ENSMUSG00000029570	0.245551559	0.62261022
Bbs4	ENSMUSG00000025235	0.146904641	0.640955185
Cdk1	ENSMUSG00000019942	0.284800077	0.643550931
Khdrbs1	ENSMUSG00000028790	0.207426672	0.668979825
Fam32a	ENSMUSG00000003039	0.148043237	0.672163069
Lmo2	ENSMUSG00000032698	0.207697932	0.713270951
Cdt1	ENSMUSG00000006585	0.10008743	0.829385623
Gadd45g	ENSMUSG00000021453	0.101362259	0.8313568
Prpf19	ENSMUSG00000024735	0.060779844	0.840012217
Tal1	ENSMUSG00000028717	0.108424283	0.851400702
Dusp3	ENSMUSG00000003518	0.133516443	0.858212043
Trp53	ENSMUSG00000059552	0.057305947	0.860277928
Prpf19	ENSMUSG00000024735	0.049440533	0.869339807
Lrp5	ENSMUSG00000024913	0.094789231	0.871833206

Rps6ka2	ENSMUSG00000023809	0.072750988	0.890207362
Gata2	ENSMUSG00000015053	0.088197621	0.891491288
Ctcfl	ENSMUSG00000070495	0.102317124	0.891742393
Gata2	ENSMUSG00000015053	0.064464943	0.919929629
Rnf167	ENSMUSG00000040746	0.04366622	0.921645502
Usp8	ENSMUSG00000027363	0.027595411	0.933608237
Psmd13	ENSMUSG00000025487	0.023589182	0.936875917
Ing4	ENSMUSG00000030330	0.02363832	0.945430275
Txnip	ENSMUSG00000038393	0.014928685	0.966993068

Supplemental Table 4. List of qPCR probes used in this study.

SL	Probe Name	Assay ID
1	Cdkn2a	Mm00494449_m1
2	Ccnd2	Mm00438070_m1
3	Ccna2	Mm00438063_m1
4	Ccne1	Mm01266311_m1
5	Cdkn1b	Mm00438168_m1
6	Yes1	Mm00501523_m1
7	Cdk7	Mm01282896_m1
